# P-2088. Correlation of pediatric blood culture transport time and time-to-positivity since microbiology service centralization

**DOI:** 10.1093/ofid/ofae631.2244

**Published:** 2025-01-29

**Authors:** Eugene Yeung

**Affiliations:** University of Ottawa

## Abstract

**Background:**

Guidance from Clinical & Laboratory Standards Institute (CLSI) and Infectious Diseases Society of America (IDSA) both recommend that transport of blood culture specimens from collection to laboratory accession should take less than 2 hours to preserve the specimen quality, such as positivity rate and time-to-positivity (TTP). This recommendation could be impractical in Canada as many diagnostic microbiology laboratories are moving towards centralization, in which microbiology services are relocated from individual laboratories to regional sites, where resources and personnel are consolidated. This is especially crucial for the pediatric population as different published guidelines are available on the required pediatric blood culture volume, which could affect their positivity rate and TTP.

**Methods:**

The current study investigated the blood culture transport time and TTP for Children’s Hospital of Eastern Ontario (CHEO), a tertiary pediatric hospital, situated about 700 meters away from Eastern Ontario Regional Laboratory Association (EORLA) microbiology laboratory, which has consolidated the microbiology services for 18 hospitals in Eastern Ontario since 2010’s. All first positive blood cultures from patients at CHEO from 1 November 2019 to 31 October 2020 were included. TTP was defined as the time from collection to a positive signal from the automated incubators. Two-tailed Spearman’s correlation was used to determine the association between transport time and TTP.

**Results:**

A mean transport time of 2.95 hours and a mean TTP of 31.8 hours were calculated from the 268 episodes of first positive blood culture from patients in a year (mean patient age 6.27 years). Only 82 specimens were transported to the laboratory in less than 2 hours. A weak but statistically significant correlation (rs = 0.17, p = 0.0047.) was observed between transport time and TTP.

**Conclusion:**

Even for a hospital within walking distance of a regional microbiology laboratory, the blood culture transport time was unable to meet the guidance recommendation, associated with delay in TTP. Centralization of microbiology services could affect the quality of critical clinical specimens. Remedial actions, such as molecular testing, could be considered to salvage specimens if they are crucial for diagnoses.

**Disclosures:**

All Authors: No reported disclosures

